# An Efficient Protocol for Single-Cell Cloning Human Pluripotent Stem Cells

**DOI:** 10.3389/fcell.2019.00011

**Published:** 2019-01-31

**Authors:** Amar M. Singh

**Affiliations:** Department of Biochemistry and Molecular Biology, Center for Molecular Medicine, University of Georgia, Athens, GA, United States

**Keywords:** pluripotent stem cells, human embryonic stem cells, induced pluripotent stem cells, single-cell, cloning protocol

## Abstract

Genomic manipulation of human pluripotent stem cells (hPSCs) has become essential to introduce genetic modifications and transgenes, and develop reporter lines. One of the major bottlenecks in gene editing is at the stage of single-cell cloning, which is thought to be variable across hPSC lines and is substantially reduced following a transfection. Due to the difficulty of performing fluorescent-assisted cell sorting (FACS) for single-cell isolation of hPSCs, previous approaches rely on manual colony picking, which is both time-consuming and labor-intensive. In this protocol, I provide a method for utilizing FACS to generate single-cell clones of hPSCs with efficiencies approaching 40% within 7–10 days. This can be achieved by sorting cells onto a feeder layer of MEFs in a stem cell defined medium with KSR and a Rock inhibitor, as early as 1–2 days following a transfection, streamlining the gene editing process. The approach described here provides a fundamental method for all researchers utilizing hPSCs for scientific studies.

## Introduction

Human pluripotent stem cells (hPSCs), including human embryonic stem cells (hESCs), and induced pluripotent stem cells (iPSCs), have become an essential cellular model system for research that span the spectrum of studies from developmental biology up through translational and therapeutic investigations (Yamanaka, 2012; Wu and Izpisua Belmonte, 2015; Rossant and Tam, 2017). These studies have been enabled, in part, due to considerable improvements in the techniques to passage and grow the cells. Original methods of maintaining hESCs were challenging, relying on manual passaging and growing the cells on feeder layers of mouse embryonic fibroblasts (MEFs), or using MEF-conditioned media (Thomson et al., 1998; Xu et al., 2001; Rosler et al., 2004). Progress was made by moving away from feeders and using extracellular matrices (such as Matrigel or Geltrex) to provide a substrate for cellular attachment (Xu et al., 2001; Brimble et al., 2004; Carpenter et al., 2004; Rosler et al., 2004). Furthermore, serum-free, defined media formulations were also developed such as mTESR (Ludwig et al., 2006), or HAIF (Wang et al., 2007; Singh et al., 2012), which is marketed as StemPRO hESC SFM by Thermo Fisher Scientific. Further refinements to media were also developed to eliminate bovine albumin and reduce media complexity by developing E8 medium (Chen et al., 2011).

Improvements to manual passaging techniques were also developed. While traditional methods of passaging cells (trypsin or collagenase) were found to result in reduced survival, karyotypical abnormalities or spontaneous differentiation in hESCs (Buzzard et al., 2004; Draper et al., 2004; Mitalipova et al., 2005), other enzymatic methods (such as using dispase or accutase) were found to be more permissive to passaging, especially when used in conjunction with a ROCK inhibitor (Watanabe et al., 2007). Non-enzymatic approaches have surfaced more recently by using EDTA-based detachment solutions (Beers et al., 2012), or products such as ReLeSR. It should be noted that non-enzymatic approaches typically are more akin to manual passaging, in that cells are passaged as “clumps,” while enzymatic approaches, such as Accutase or TrypLE Select, permit single-cell disassociation prior to seeding of the cells. Overall, these improvements in growing and passaging hPSCs have facilitated the use of these cells for numerous research disciplines.

One of the major challenges for hPSC researchers that still remains is the single-cell cloning of hPSCs at a high efficiency, especially when following a transfection. When only a few clones are sufficient, manual colony-picking has proven to be an effective approach. However, when >50 single-cell clones are needed, such as when genetically modifying the cells, manual colony picking is not ideal as the process is both labor-intensive and inefficient. Here, I provide a detailed procedure for the high efficiency single-cell cloning of hPSCs. This procedure significantly improves current methods for clonal isolation of hPSCs for gene editing studies.

## Materials and Equipment

### Reagents

1. MEFs (EMD Millipore, PMEF-N).
2. DMEM (Corning, 10014CV).
3. ES-qualified FBS (Atlanta Biologicals, S10250).
4. Antibiotic-Antimycotic (Corning, 30004CI).
5. GlutaGRO (Corning, 25015CI).
6. MEM non-essential amino acids (Gibco, 11140050).
7. 2-mercaptoethanol (BME; Gibco, 21985023).
8. 0.2% gelatin (Sigma, G1393).
9. hPSC Defined Medium (DM); (mTeSR1 – StemCell Technologies, 85850; StemPro hESC SFM – Thermo Fisher Scientific, A1000701; E8 – StemCell Technologies, 05990).
10. KnockOut Serum Replacement (KSR; Thermo Fisher Scientific, 10828028).
11. ROCK inhibitors (RevitaCell – Thermo Fisher Scientific, A2644501; Y27632 – R&D Systems, 1254/10).
12. Basement membrane matrices (Geltrex – Thermo Fisher Scientific, A1413302; Cultrex – R&D Systems, 3434-010-02; Matrigel – Corning, 354277).
13. Accutase (Innovative Cell Technologies, AT104).
14. Dulbecco’s Phosphate Buffered Salt Solution (DPBS), calcium and magnesium free (Corning, 21031CM).
15. 96-well cell culture plates (Corning, 353072).
16. Fifteen milliliters conical centrifuge tubes (Corning, 430790).
17. Flow cytometry tube with cell strainer cap (Corning, 352235).

### Equipment

1. Fluorescent assisted cell sorter (Beckman Coulter, MoFlo Astrios EQ).
2. Centrifuge (Thermo Fisher Scientific, Accuspin 1R), with a swinging bucket rotor and adapters capable of spinning conical tube and 96-well plates.
3. Tissue culture incubator at 37°C, 5% CO_2_ (Sanyo, MCO20A1C).
4. Biological Safety Cabinet (Thermo Fisher Scientific, Labconco A2).
5. Inverted light microscope (Leica DM IL).
6. Multi-channel aspirator (Corning, 4930 and 4931).
7. 12-channel pipettor (Rainin, 17013808).

### Media Formulations

#### 0.2% Gelatin (1 L)

Warm gelatin at 37°C, until solubilized. Add 100 ml of gelatin to 900 ml of DPBS and filter sterilize. Store at 4°C.

#### MEF Media (500 ml)

Fifty milliliters ES-qualified FBS, 5 ml GlutaGRO, 5 ml penicillin/ streptomycin, 5 ml minimal non-essential amino acids, 500 μl of bME, 435 ml DMEM.

#### Defined Medium (DM); 500 ml

Hundred milliliters mTeSR 5× supplement, 5 ml antibiotic/ antimycotic solution, 395 ml of basal mTeSR medium.

*Note*: Other DM may be used, such as E8 or StemPRO hESC SFM.

#### Single-Cell Cloning (SCC) Medium; 500 ml

Hundred milliliters mTeSR 5× supplement, 5 ml penicillin/ streptomycin, 50 ml KSR, 345 ml of basal mTeSR medium.

*Note*: SCC medium can be used with E8 or StemPRO, by using their corresponding supplement formulations. Dilute the supplement as appropriate to obtain a 1× final concentration.

## Procedure

**FIGURE 1:**
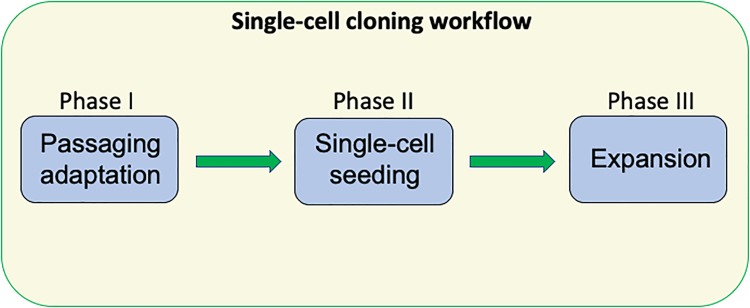
The overall scheme for efficient single-cloning of hPSCs. This approach consists of three phases and routinely results in efficiencies of ∼40%.

This method for single-cell cloning consists of three stages (Figure 1). In the first stage, you will prepare you cells for single-cell cloning, which is required prior to any genetic manipulation of the cells, such as a transfection or transduction. In the second stage, you will perform the seeding of single-cells into 96-well plates. In the third stage, you will perform passaging and expansion as needed for your subsequent experimental plans.

### Stage 1: Adapting hPSCs to Single-Cell Passaging

Day 1:

1. Pre-coat a 60 mm cell culture dish with 2 ml of Geltrex (or equivalent ECM, such as Cultrex or Matrigel) at a 1/200 dilution for a minimum of 30 min in a 37°C cell culture incubator.
2. Passage your cells using your standard approach, maintaining 1 plate of cells under your current conditions, and a 2nd plate that has been pre-coated with Geltrex in your defined media (DM), such as mTesr1, StemPRO, or E8. Seeding density should be at your normal range, such as a 1:5–1:10 ratio if clump passaging. The first plate will be used simply for maintaining your cells under current conditions, while the second plate will be used for adapting your cells to single-cell passaging.

Days 2–4:

3. Grow the hPSCs for 3–4 days with daily media changes in your DM, until colonies are of a large size (∼500 μm diameter), but not quite touching and have minimal differentiation. If necessary, remove any differentiated cells by scraping with a needle or pipet tip.

Day 5:

4. Aspirate media from cells and wash cells once with DPBS.
5. Add 2 ml of room temperature Accutase to the plate and return plate to 37°C incubator for 3 min, or until cells have detached.
6. Add 2 ml of DPBS to the plate, transfer to a 15 ml conical tube and centrifuge at 1000 rpm for 4 min.
7. Aspirate supernatant and add 1 ml of fresh DM and resuspend the cell pellet.
8. Aspirate Geltrex from a new 60 mm plate and add 3 ml of DM with 1× RevitaCell to the plate.
9. Add 200 μl from the cell suspension (1:5 ratio) to the new plate containing DM+RevitaCell.

*Note*: RevitaCell should only be used when initially seeding cells. Y27632 may be used in place of RevitaCell, however, cell viability and survival are much lower. Cells may also have an abnormal morphology in Y27632 – less circular and more spindle-shaped; however, they will still be fully pluripotent and retain pluripotency markers.

Days 6–10:

10. Change media on the plate daily to DM only and grow for 3–4 days until clones are near confluent.

Days 11–20:

11. Repeat steps 4–10, 2 more times, so that cells are fully acclimated to single-cell passaging.
12. Cells may now be used for any transfection or transduction protocol that you require for your experimental needs, and you may perform single-cell sorting 1–2 days following the transfection. We recommend that transfection be optimized for efficiency and viability for each cell line that you use, prior to experimentation (see section “Anticipated Results”).

### Stage 2: Single-Cell Seeding

Day 1:

1. Add 0.2% gelatin to 2–4 96-well plates (100 μl/well) for 15 min at room temperature.
2. Thaw MEFs and resuspend at a concentration of 1 million cells per 10 ml of MEF medium (10 ml per 96-well plate).
3. Aspirate gelatin from plates.
4. Add MEFs to plate (100 μl/well).
5. Incubate overnight at 37°C, 5% CO_2_.

Day 2:

6. Aspirate media from hESCs/hiPSCs and wash with D-PBS.
7. Add room temperature Accutase (5 ml for 100 mm dish) and incubate for 3–5 min at 37°C (until cells are dissociated).
8. Add an equal volume of D-PBS (5 ml for 100 mm dish), transfer to a 15 ml conical tube and pellet cells.
9. Resuspend cell pellet in SCC media with RevitaCell (1–2 million cells/ml), and transfer to flow tube.
10. Aspirate media from 96-well plates, add SCC media with RevitaCell.

*Note*: Addition of KSR to SCC media is absolutely essential for successful cloning, as it keeps MEFs alive in the stem cell defined medium and dramatically improves cloning efficiency. When preparing media, reduce basal media volume (DMEM-F12) to account for increased volume from KSR. Avoid reducing growth factor concentrations (e.g., do not simply add 10% KSR to mTESR1 complete medium, as this would dilute bFGF and TGF-β).

11. Utilize fluorescent-assisted cell sorting (FACS) to sort 1 cell/well into 96-well plates.

*Note*: As an alternative approach, limiting dilution may be used where cells can be diluted to 50–100 cells in 10 ml, and 100 μl be pipetted into each well of a 96-well plate.

12. Return cells to incubator.

Days 3–9:

13. Aspirate media from plate.
14. Add fresh SCC media (do NOT include RevitaCell).
15. Continue to change media every other day up until day 7, and then change media daily until PSC colonies are of sufficient size.
16. Starting at day 9 (7 days after FACS), evaluate plates for number of colonies and colony size. Draw circles around wells with 1 colony only.

*Note*: As colonies are being grown on MEFs, if clones become overgrown there will be an increased tendency for unwanted differentiation. Minor amounts of differentiation are usually not problematic, and will be reduced during Accutase passaging.

### Stage 3: Passaging and Expansion

Day 12 (approximately, depending on colony size):

17. Geltrex coat 1–2 96 well plates, depending on the number of colonies, incubate for 30 min at 37°C.
18. Wash 96-well plates containing PSC colonies in DPBS and add Accutase (50 μl/well) to colony-containing wells, return to incubator until colonies are detached.

*Note*: Larger colonies take a longer to detach – carefully view cell detachment under a microscope.

19. Add 150 μl of DM to the wells containing Accutase.

*Note*: Do not use DPBS instead of DM, cell will attach better to the plate during centrifugation in DM than DPBS.

20. Aspirate geltrex from 96-well coated plates and transfer cell suspension from each well to an independent well. Centrifuge entire plate at 1000 rpm for 4 min. Visualize that cell have successfully attached to wells.
21. Very carefully aspirate media from wells, being sure not to dislodge cells and add fresh DM with RevitaCell.

Days 13–18:

22. Change medium daily with DM only.
23. When wells are confluent, Accutase passage cells as before (steps 17–21 above) and transfer cells using 1/5 ratio (i.e., 40 μl of the a 200 μl cell suspension) to the new plate.
24. Transfer the remaining 160 μl of your cell suspension to a 96-well plate for your downstream analysis, such as genotyping by PCR. Cells maybe be pelleted and stored in -20°C for collecting genomic DNA.
25. After genotyping is complete. Correct clones should expanded first to 24-well plate, then to 6-well plate, and cells should be frozen for long-term storage in liquid N_2_ as soon as possible.

## Anticipated Results

**FIGURE 2:**
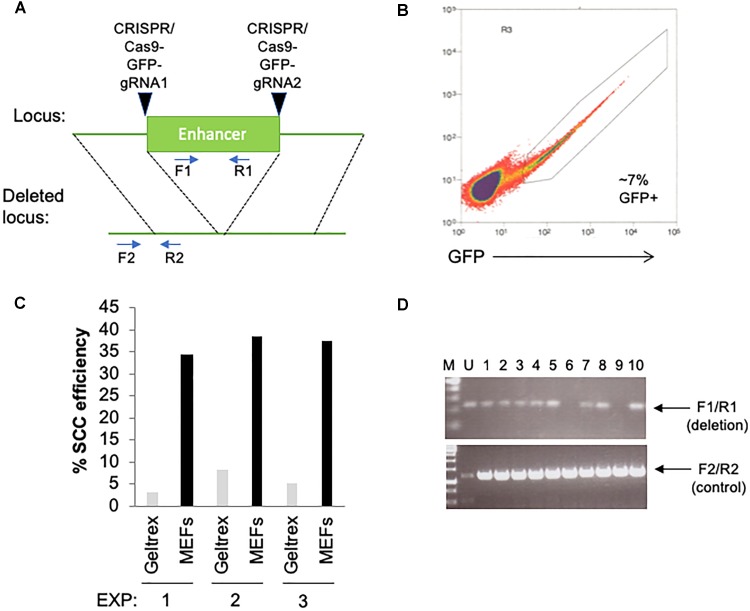
Utilization of feeder layers are more efficient than basement membrane matrices when single-cell cloning hPSCs with RevitaCell and KSR following a transfection. **(A)** Diagram depicting the targeting site for each CRISPR/Cas9 GFP plasmids and predicted results following genomic deletion. Primer sequences are indicated by arrows. **(B)** Two days following an electroporation of the CRISPR/Cas9 plasmids, hPSCs were seeded by FACS as single-cells onto 96-well plates coated with Geltrex (1/100 dilution) or pre-seeded with MEFs (1 million cells/96-well plate) using three replicate conditions. **(C)** Single-cell cloning (SCC) efficiency following the FACS isolation of transfected cells. **(D)** Genomic screening by PCR using indicated primers to identify the deletion of the enhancer.

We have been using this procedure extensively for clonal isolation of hPSC following genomic manipulation, as early as 1–2 days following a transfection for several research projects including gene knockouts, reporter knock-in and developing stable, ectopically expressed cell lines. In one example, we have used this approach to delete a putative enhancer of interest (Figure 2). Two CRISPR/Cas9-GFP plasmids (Addgene, PX458) containing gRNAs that target each side of a putative enhancer were transfected by electroporation (Neon Transfection System, Thermo Fisher Scientific) into hPSCs (Figure 2A). Cells were subsequently sorted at 2-days post-transfection by GFP onto 96-well plates containing MEFs or Geltrex (Figure 2B,C). Using the above described procedure, we obtained single-cell cloning efficiencies ranging from 35 to 40% on MEFs. After subsequent colony expansion, 10 clones were screened for deletion of the enhancer and compared to the untransfected control, of which 2 clones exhibited bi-allelic enhancer deletion (Figure 2D). In general, we have also found the results of this single-cell cloning approach to be largely comparable using independent lines of hESCs and iPSCs. In total, we have used this method on >10 hPSC lines including common lines such as WA09 and K3 iPSCs, along with in-house developed iPSC lines.

The major improvement with this protocol over others is in the use of a MEF feeder layer with a defined media plus KSR supplementation, along with RevitaCell, during the single-cell sorting following a transfection. We have been able to obtain roughly 3–4 times higher efficiency with this protocol using MEF feeder layers, than that with a DM plus RevitaCell on Geltrex (Figure 2C). This indicates that in the absence of a MEF feeder layer, single-cell cloning efficiency, specifically following a transfection, will be considerably reduced. Indeed we have found higher single-cell cloning efficiencies in un-transfected cells (>25%) on Geltrex plus RevitaCell alone. While other studies have suggested efficiencies ranging from 30 to 40% using a DM with RevitaCell on Geltrex (Chen and Pruett-Miller, 2018), these studies do not take into account the reduced viability following transfections by electroporation or lipofection. Thus, our procedure of sorting onto MEFs in an hPSC-DM and KSR with a Rock inhibitor overcomes the obstacle of reduced viability following a transfection to facilitate high-efficiency single-cell cloning.

We have experimented with several different transfection reagents including, electroporation using the Neon (Life Technologies), Amaxa Nucleofector II and 4D-Nucleofector; and lipofection using Lipofectamine-3000 (Thermo Fisher Scientific), Lipofectamine Stem Reagent (Thermo Fisher Scientific), and TransIT-2020 (Mirus Bio). In all cases, we have noticed a trade-off between transfection efficiency and cell viability, with higher transfection efficiencies resulting in lower viability for several different hESC and iPSC lines. We have found that cell viability is consistently reduced following these transfection approaches compared to un-transfected cells, and always results in lower single-cell cloning efficiency on Geltrex or Matrigel than on MEFs. Using the Neon electroporation system with 2 million cells and 8 μg of DNA (1050v pulse voltage, 30 ms pulse width, 2 pulses) we were able to obtain 7% GFP positive cells (Figure 2C) with a homemade hPSC line. We have found that transfection efficiencies must be optimized for each hPSC line. For example, with the hPSC line used in Figure 2, electroporation was far more efficient than lipofection, the latter of which only resulted in 1–2% efficiency with the Lipofectamine Stem Reagent. However, with WA09 (H9) hESCs we have routinely obtained 20% transfection efficiency, using the Neon system, but >30% with Lipofectamine Stem Reagent. Overall, from using the Neon electroporation method with CRISPR/Cas9 plasmids, and this single-cell cloning procedure, we were able to obtain a DNA editing efficiency of 20% (Figure 2D).

Another major advantage of our protocol is its versatility. A user can use any defined medium that they currently use with their cells, and then supplement that with KSR plus RevitaCell to allow for single-cell sorting onto MEFs. We have successfully used our approach in different types of defined media including mTeSR1, E8, and STEMPRO hESC SFM, without noticing any major differences in cloning efficiencies. This provides a cost-reduction that would be associated with buying different types of defined media. Furthermore, by using a stem cell defined media as their “base growth conditions,” this enables researchers to switch their cells from different attachment substrates (MEFs, Geltrex, etc.) with ease. As indicated by our protocol, the hPSCs are moved off from MEFs and back onto Geltrex as soon as the individual colonies are of sufficiency size (<2 weeks).

A final advantage of our system is the ability to avoid manual picking of colonies following a transfection. Manual picking of 50–100 colonies could take as much as an hour or more, especially for inexperienced users, while clonal isolation by FACS can be done in minutes. While manual passaging of hPSCs is not a common procedure anymore, manual picking of transfected clones still represents a common approach, especially for gene editing applications (Blair et al., 2016; Kwart et al., 2017; Wang et al., 2017; Howden et al., 2018; Tidball et al., 2018). By using this approach, single-cell sorting hPSCs by FACS following a transfection can be done with ease and clones may be expanded for genomic screening.

One caveat to this method is with the use of feeders, and particularly MEFs. Previous reports have suggested that culturing hESCs on MEFs may have negative consequences, as MEFs may be contaminated with retroviruses (Cobo et al., 2008). Furthermore, the use of MEFs, although only transiently, does not lend itself to a xenogeneic-free methodologies, which may not be ideal for therapeutic utility of hPSCs. Although, we have not tested human cells for feeders, previous reports have suggested that human fibroblasts provide superior feeder layers to MEFs (Cobo et al., 2008; Mali et al., 2008). Human fibroblasts may therefore provide a useful cell type for single-cell cloning applications that necessitate xeno-free conditions.

Our findings that hPSCs survive better on MEFs than on basement membranes alone, such as Geltrex, even in the presence of ROCK inhibitors, raises the question of why this might be the case. One explanation for this is a “neighbor model,” where hPSCs survive better with neighboring cells than in isolation. In support of this, was the long-standing notion that hPSCs must be passaged as “clumps,” using manual passaging, collagenase, or more recently using non-enzymatic EDTA-based approaches (Chen et al., 2011), for optimal cell adherence and survival. The addition of ROCK inhibitor greatly improved the adherence of hESCs, preventing apoptosis, following single-cell passaging (Watanabe et al., 2007). We have found that single-cell cloning efficiency can still be low with ROCK inhibitor, however, following a transfection, but can be significantly improved when MEFs are present. The “neighbor model” suggests that survival signals beyond immediate adherence may be needed for a single cell to propagate and form a colony (Phadnis et al., 2015). These signals may include the sensing of neighboring cells and cytokines/chemokines, along with subsequent migration (Phadnis et al., 2015). We speculate that the MEF feeder layer used in our system provide additional survival signals, akin to neighboring hPSCs, beyond those provided by the ECM/ROCK inhibitor combination, thereby improving single-cell cloning outcomes, especially when cell viability is reduced following a transfection.

In sum, we have described a single-cell cloning procedure for hPSCs that enables high efficiency following a transfection, that is simple and straightforward to use, and that does not necessitate significant cost increases, such as the purchase of new equipment. This protocol should therefore result in enhanced research workflows for all pluripotent stem cell researchers.

## Author Contributions

AS performed the experiments, analyzed the results, developed the protocol, and wrote the manuscript.

## Conflict of Interest Statement

The author declares that the research was conducted in the absence of any commercial or financial relationships that could be construed as a potential conflict of interest.
